# Noninvasive Assessment of Prehepatic Portal Hypertension: Pilot Data on Spleen Stiffness in Portal Vein Thrombosis

**DOI:** 10.1155/ijh/5531253

**Published:** 2025-12-22

**Authors:** Kelly Tin Long Chan, Oi Ling Chan, Yin Yee Lam, Siu Wing Lawrence Lai

**Affiliations:** ^1^ Department of Medicine and Geriatrics, Pok Oi Hospital, Yuen Long, Hong Kong, pokoi.org.hk; ^2^ Department of Radiology, Tuen Mun Hospital, Tuen Mun, Hong Kong, ha.org.hk

**Keywords:** portal hypertension, portal vein thrombosis, spleen stiffness

## Abstract

**Background:**

Portal vein thrombosis (PVT) is a vascular liver disorder defined by a thrombus in the portal vein or its intrahepatic branches. Computed tomography (CT) and upper endoscopy are, respectively, used to monitor for PVT progression and portal hypertensive complications. A noninvasive modality—spleen stiffness (SS)—has shown promise in identifying clinically significant portal hypertension (CSPH) in cirrhosis. It is unknown whether SS demonstrates any correlation with PVT, a condition associated with prehepatic portal hypertension.

**Aims:**

The primary aim was to determine the association between SS and the presence of PVT. The secondary aim was to evaluate the association between SS and portal hypertension–related complications among patients with PVT.

**Methods:**

This cross‐sectional study was undertaken at two regional hospitals in Hong Kong from January 2023 to March 2024. Patients were identified via CT and allocated to either the PVT or non‐PVT group. Chronic liver disease was an exclusion criterion for both groups. SS was assessed using transient elastography within 3 months of PVT diagnosis. Demographic, clinical, and laboratory data were collected within 3 months of PVT diagnosis.

**Results:**

A total of 46 patients with PVT (median age, 69 years; interquartile range [IQR], 62–78; 74% male) were compared with 45 controls. Both SS and liver stiffness (LS) were significantly higher in the PVT cohort than in controls (SS: 27.9 [IQR, 19.5–42.8] vs. 16.9 kPa [IQR, 13.6–21.2], *p* < 0.001; LS: 6.0 [IQR, 4.8–8.6] vs. 4.6 kPa [IQR, 3.7–5.9], *p* < 0.001). Among patients with PVT, those with portal hypertension–related complications demonstrated markedly elevated SS compared with those without complications (77.7 [IQR, 47.2–85.0] vs. 24.4 kPa [IQR, 18.9–37.1], *p* < 0.001). Furthermore, SS values increased in proportion to the anatomical extent of PVT involvement.

**Conclusion:**

Elevated SS was observed in patients with PVT, particularly in those with PVT‐induced portal hypertension. Large‐scale, prospective studies are warranted to confirm the association between SS and PVT and to establish its potential role in noncirrhotic portal hypertension.

## 1. Introduction

Portal vein thrombosis (PVT) is a vascular liver disorder defined by a solid material in the portal vein or its intrahepatic branches. Monitoring of PVT with various imaging modalities such as contrast‐enhanced ultrasonography, computed tomography (CT), or magnetic resonance imaging is used to monitor treatment response [1]. Endoscopic surveillance is also required for portal hypertensive complications. Further investigation of alternative noninvasive and cost‐effective modalities in monitoring PVT progression and portal hypertension should be explored.

Various noninvasive modalities have emerged over the years to assess clinically significant portal hypertension (CSPH) in patients with cirrhosis, including the use of transient elastography (TE) to measure liver stiffness (LS) and spleen stiffness (SS) [2–4]. SS has emerged as an adjunct to LS in (1) identifying the presence of CSPH [5–7], (2) potentially assessing treatment response to nonselective beta‐blockers in CSPH [8, 9], and (3) stratifying risk for hepatocellular carcinoma recurrence for patients with chronic liver disease [10]. SS in combination with LS and platelet count improves the sensitivity and specificity in identifying CSPH [11, 12].

Available data regarding whether SS is increased in PVT and associated portal hypertension are scarce and show conflicting results [13–15]. Sharma et al. [14] evaluated 65 patients with extrahepatic portal vein obstruction (EHPVO), demonstrating higher LS and SS in the PVT group compared with the control group. For SS, a cutoff of 20.9 kPa for identifying PVT resulted in a sensitivity of 93% and a specificity of 100%. Patients with history of clinical bleeding had higher SS compared with the nonbleeding group (mean 60.4 vs. 30.3 kPa).

This finding is in contrast with the study by Madhusudhan et al. [15], in which 21 EHPVO patients were evaluated. All patients received variceal screening by endoscopy, SS measurement by 2D‐SWE, and intraoperative portal pressure measurement via the omental vein during splenorenal shunt surgery. The study demonstrated a correlation between portal pressure and severity of varices, but no correlation was found between SS and portal pressure, duration of disease, or grading of endoscopic varices.

This study was a prospectively recruited cross‐sectional cohort. The primary aim of this study was to evaluate whether patients with noncirrhotic PVT had higher SS than controls. The secondary aim was to evaluate whether noncirrhotic PVT patients with portal hypertension had higher SS than those without.

## 2. Materials and Methods

### 2.1. Study Design

The study included two groups of patients: those with PVT and those without PVT. PVT diagnosis was made by CT. All patients were seen in the clinic or hospital ward with written informed consent obtained. TE was performed within 3 months of PVT diagnosis (by CT). The study, approved by the Central Institutional Review Board Ethics Committee of Hospital Authority, Hong Kong, was conducted in two regional hospitals in the New Territories West in the period of January 2023 to March 2024.

Patients with PVT were retrieved via the radiology information system (RIS) using the keyword “portal vein thrombosis.” Inclusion criteria included those with thrombus in the vessel lumen, with or without involvement of the splenic vein or superior mesenteric vein. Patients were excluded if they had the following: chronic liver disease (defined by viral hepatitis, steatotic liver disease, and autoimmune liver disease), cirrhosis (defined by radiological features of cirrhosis or LS above 15 kPa [16–18]), hepatobiliary or pancreatic malignancy, gross ascites, uncooperative with examination, deceased before recruitment, below 18 years old, pregnant, or refusal to participate. Patients with chronic liver disease and cirrhosis were excluded to determine the isolated effect of PVT on SS.

Controls were retrieved using the Clinical Data Analysis and Reporting System (CDARS) if they had CT abdomen performed within the past 3 months and met the following criteria: no structural lesions (including PVT) in the liver on CT imaging, no history of chronic liver disease (defined above), negative serology for hepatitis B and C, and normal liver function.

### 2.2. TE Examination

TE examination was performed within 3 months of PVT diagnosis by CT, using a FibroScan Expert 630 model (Echosens, Paris, France) at Pok Oi Hospital by trained staff. Patients were asked to fast for at least 3 h before examination. For LS measurement, the patient was positioned in dorsal decubitus with their right arm in maximal abduction. The liver‐dedicated probe (at 50 Hz) was perpendicularly placed at the right midaxillary line, at the level of the xyphoid process, between the intercostal space. Measurements were considered valid if at least 10 consecutive measurements were obtained at the same site within an IQR/median of < 30% [19]. The patient was then repositioned by rotating 180° while remaining in a supine position, with their left arm in maximal abduction. The ultrasound probe (using B mode) was placed at the left posterior axillary line, at the level of the xyphoid process. The spleen was localized; then, marking was performed. The spleen‐dedicated probe at 100 Hz was placed perpendicular to the marking site with measurement performed. Measurements were considered valid if at least 10 consecutive measurements were obtained at the same site within an IQR/median of < 30%.

### 2.3. Clinical and Laboratory Evaluation

Baseline demographics, clinical information, and laboratory parameters were obtained within 3 months of PVT diagnosis (by CT).

### 2.4. Definition of Portal Hypertension

Portal hypertension was defined by either varices on endoscopy or portosystemic collaterals or varices on CT imaging [20].

### 2.5. Statistical Analysis

The sample size was calculated based on diagnostic accuracy. The minimum sensitivity of SS in detecting PVT was set at 75% (with power of 80% and *α* = 0.05); hence, 42 patients with PVT were needed to be included to ensure the lower limit of the one‐sided confidence interval for sensitivity is at least 0.75.

Statistical analysis was performed using SPSS Version 26.0. Continuous variables were reported as median (with interquartile range). Categorical variables were expressed as counts with percentages. For group comparisons, the Wilcoxon rank‐sum Mann–Whitney *U* test or the Kruskal–Wallis method was used for continuous variables, whereas the chi‐square test was used for categorical variables. Correlation analysis of SS and other parameters was calculated using the Spearman correlation coefficient (*r*_*s*_). Multiple linear regression analysis with backward stepwise selection was performed to analyze independent predictors of SS. *p* value of < 0.05 was considered statistically significant.

## 3. Results

### 3.1. Case Identification

In the PVT group, 239 patients were retrieved from the RIS database in Tuen Mun Hospital and Pok Oi Hospital from January 2023 to March 2024. One hundred ninety patients were excluded: 12 with cirrhosis, 73 with hepatobiliary malignancy, 4 with pancreatic malignancy, 15 with liver metastasis, 3 with gross ascites, 1 uncooperative patient, 73 deceased before recruitment, and 10 who refused to enter study. Forty‐nine patients were enrolled in the study. LS and SS measurements by TE were performed in all patients. Two patients had invalid SS measurements due to high body mass index (BMI) and splenic atrophy and were excluded. Hence, 46 patients with PVT and fulfilling all inclusion criteria were enrolled.

**Figure 1:**
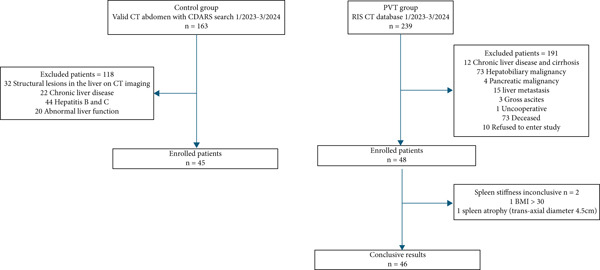
Flow chart of studied patients. BMI = body mass index, CDARS = Clinical Data Analysis and Reporting System; CT = computer tomography; RIS = radiology information system.

In the control group, 163 patients with CT abdomen performed within 3 months of the study period were retrieved using CDARS. One hundred eighteen patients were excluded according to the exclusion criteria. Forty‐five patients with negative CT findings were enrolled in the control group (Figure 1).

### 3.2. Demographic Characteristics

Table 1 summarizes the baseline characteristics of the 91 patients enrolled in the study. The median age was 69 years and 74% male. Almost half of the PVT patients (45.7%) had a branch type thrombus. The predominant etiology was intra‐abdominal inflammation (cholangitis, cholecystitis, liver abscess, and pancreatitis). The median ALT, bilirubin count, and spleen length were higher in the PVT group, whereas the albumin level was lower in the PVT group. These differences were statistically significant. Anticoagulants were prescribed to 52.2% of patients. The mean time of TE was performed 43 days following PVT diagnosis in CT.

**Table 1:** Demographic data of patients.

	**PVT (** **n** = 46 **)**	**Control (** **n** = 45 **)**	**p**
Demographic			
Age, median (IQR), years	69 (62–78)	64 (57–74)	0.069
Male, *n* (%)	34 (74%)	27 (60%)	0.158
Body mass index, median (IQR), kg/m^2^	24.1 (21.9–26.2)	22.8 (20.7–26.4)	0.337
Laboratory parameters			
ALT, median (IQR), IU/L	27.0 (15.8–44.3)	19.0 (12.0–24.5)	0.005
Bilirubin, median (IQR), *μ*mol/L	12.5 (8.9–19.0)	8.0 (6.0–11.0)	< 0.001
Platelet count, median (IQR), 10^9^/L	212 (157–326)	240 (177–282)	0.614
Albumin, median (IQR), g/L	37.0 (30.8–41.3)	43.0 (40.5–45.0)	< 0.001
Creatinine, median (IQR), *μ*mol/L	80.0 (66.3–96.0)	79.0 (60.5–91.5)	0.700
Radiological			
Spleen length, median (IQR), cm	10.0 (8.4–11.4)	8.7(7.6–10.6)	0.024
Characteristics of PVT			
Anatomical distribution			
Intrahepatic	9 (19.6%)		
Branch	21 (45.7%)		
Main	8 (17.4%)		
Extensive (into SMV/splenic vein)	8 (17.4%)		
Additional splanchnic thrombus			
Only splenic vein	1 (2.17%)		
Only SMV	5 (10.9%)		
Both SMV and splenic vein	2 (4.35%)		
Etiology			
Cholangitis	21 (45.7%)		
Cholecystitis	4 (8.70%)		
Liver abscess	6 (13.0%)		
Hematological^a^	2 (4.35%)		
Pancreatitis	4 (8.70%)		
Idiopathic	6 (13.0%)		
Appendicitis	1 (2.17%)		
Hepatitis	1 (2.17%)		
Intestinal obstruction	1 (2.17%)		
Evidence of portal hypertension			
Yes	6 (13.0%)		
No	40 (87.0%)		
Endoscopic			
Varices^b^			
Yes	4 (14.3%)		
Low‐grade varices	4		
High‐grade varices	0		
No	24 (85.7%)		
Clinical			
Variceal bleeding after PVT onset			
Yes	0 (0%)		
No	100 (100%)		
Use of anticoagulation			
Yes	24 (52.2%)		
No	22 (47.8%)		
Use of nonselective beta‐blocker			
Yes	7 (15.2%)		
No	39 (84.8%)		

Abbreviations: ALT, alanine transaminase; IQR, interquartile range; PVT, portal vein thrombosis; SMV, superior mesenteric vein.

^a^Essential thrombocytosis and protein C deficiency.

^b^Eighteen patients did not undergo upper endoscopy performed within 6 months of PVT diagnosis.

Only 13% of the PVT patients had portal hypertension. None of the patients had variceal bleeding between the diagnosis of PVT and TE examination. Eighteen (39.1%) patients did not undergo upper endoscopy within 6 months. Of those that did have upper endoscopy performed, four patients had low‐grade varices. It was noted that of the seven patients taking nonselective beta‐blockers, one did not receive it for the indication of portal hypertension.

A total of 24 patients (52%) were initiated on anticoagulation therapy; of these, seven received warfarin and 16 were prescribed a novel oral anticoagulant (apixaban or edoxaban). The comparatively low proportion of patients receiving anticoagulation was attributable to multiple factors: (i) patient‐related concerns regarding bleeding risk; (ii) financial constraints, with some patients declining therapy as they did not wish to self‐finance a novel oral anticoagulant and were unwilling to take warfarin; and (iii) physician judgment that anticoagulation would confer limited benefit in patients with isolated intrahepatic PVT involvement or an underlying reversible etiology. The median interval between the date of anticoagulation therapy initiation and SS was 54 days.

### 3.3. Comparing LS and SS in PVT Versus Control Group

**Figure 2:**
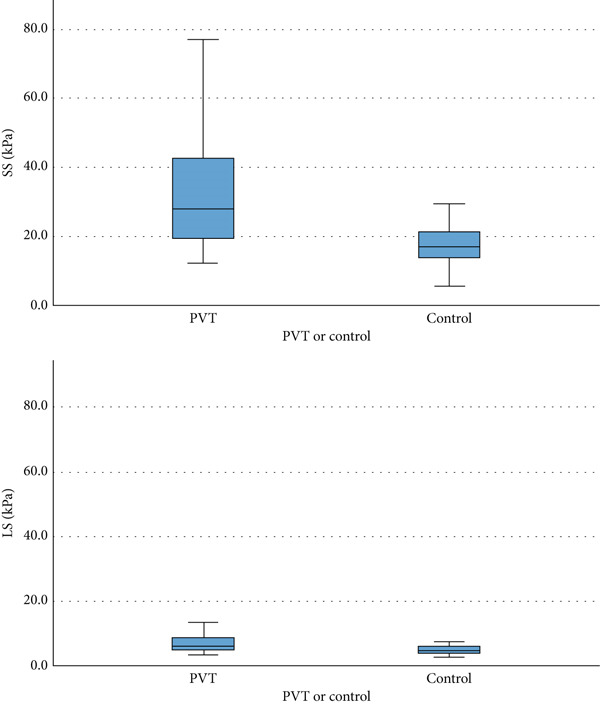
Graph comparing SS and LS in the PVT group versus the control group. LS = liver stiffness; PVT = portal vein thrombosis; SS = spleen stiffness.

There was a statistically significant difference in the median SS (27.9 [19.5–42.8] vs. 16.9 [13.6–21.2] kPa, *p* < 0.001) and median LS (6.0 [4.8–8.6] vs. 4.6 [3.7–5.9] kPa, *p* < 0.001) between the PVT and the control groups (Figure 2).

### 3.4. Sensitivity, Specificity, Positive Predictive Value, and Negative Predictive Value of SS and LS for PVT Detection

**Figure 3:**
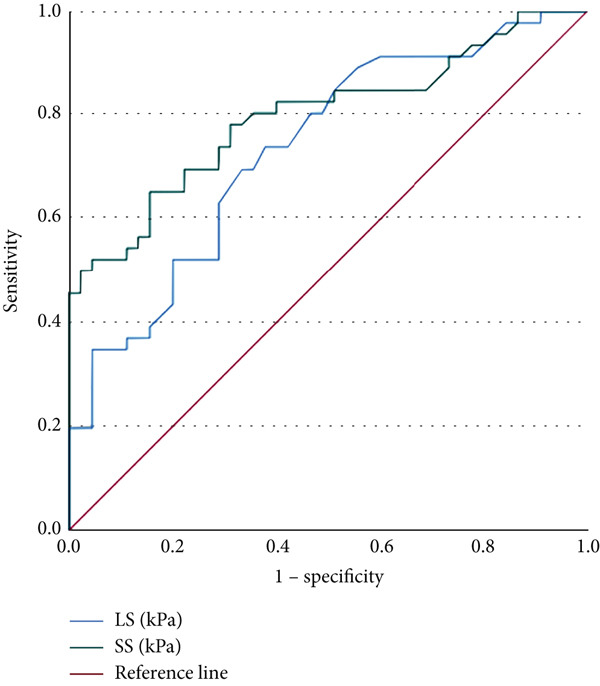
Receiver‐operating characteristic curves for SS and LS for PVT. LS = liver stiffness; SS = spleen stiffness.

At a cutoff of 19.4 kPa using the Youden index, SS had sensitivity of 78.3%, specificity of 68.9%, positive predictive value of 72.0%, and negative predictive value of 75.6% for discriminating between patients with and without PVT. The area under the receiver‐operating characteristic curve (AUC) was 0.798 (95% CI 0.705–0.890) (*p* = 0.04). At a cutoff of 5.3 kPa, LS had sensitivity of 69.9%, specificity of 66.7%, positive predictive of 68.1%, and negative predictive value of 68.2% in discriminating between patients with and without PVT. The AUC was 0.736 (95% CI 0.634–0.837) (*p* = 0.05) (Figure 3).

### 3.5. Comparing Patients With and Without Portal Hypertension in PVT

Patients with and without portal hypertension in the PVT group were compared. Patients with portal hypertension had higher SS (77.7 [47.2–85.0] vs. 24.4 [18.9–37.1]) kPa, *p* < 0.001) and longer spleen length (13.2 [12.0–15.3] vs. 9.9 [8.3–11.0] cm, *p* < 0.001) (Table 2). There was no statistical difference of LS (5.9 [4.9–9.8] vs. 5.8 [4.8–8.4] kPa, *p* = 0.937), platelet count (136 [116–278] vs. 217 [165–358] × 10^9^/L, *p* = 0.07), bilirubin (17 [12–28] vs. 12 [8–17] *μ*mol/L, *p* = 0.160).

**Table 2:** Comparing SS, LS, and laboratory parameters in PVT with or without portal hypertension.

	**Patients with portal hypertension (** **n** = 6 **)**	**Patients without portal hypertension (** **n** = 40 **)**	**p**
SS (kPa)	77.7 (47.2–85.0)	24.4 (18.9–37.1)	< 0.001
LS (kPa)	5.9 (4.9–9.8)	5.8 (4.8–8.4)	0.937
ALT, median (IQR), IU/L	26 (17–43)	28 (13–44)	0.939
Platelet count, median (IQR), 10^9^/L	136 (116–278)	217 (165–358)	0.07
Bilirubin, median (IQR), *μ*mol/L	17 (12–28)	12 (8–17)	0.160
Spleen length (cm)	13.2 (12.0–15.3)	9.9 (8.3–11.0)	< 0.001

Abbreviations: ALT, alanine transaminase; LS, liver stiffness; SS, spleen stiffness; PVT, portal vein thrombosis.

### 3.6. Sensitivity, Specificity, Positive Predictive Value, and Negative Predictive Value of SS for Portal Hypertension in PVT

At a cutoff of 29.3 kPa using the Youden index, SS had sensitivity of 100%, specificity of 62.5%, positive predictive value of 28.6%, and negative predictive value of 100% for discriminating between patients with and without portal hypertension in PVT. The AUC was 0.91 (95% CI 0.791–1.000) (*p* = 0.001).

### 3.7. Correlation of SS and Other Parameters in Patients With PVT Group

There was significant correlation between SS and LS (*r*_*s*_ = 0.45, *p* = 0.002) and spleen length (*r*_*s*_ = 0.40, *p* = 0.006), whereas there was no significant correlation between SS and platelet, bilirubin, age, and BMI (Table 3). To determine independent variables affecting SS, a multivariate regression model was used. Covariates with *p* < 0.15 in univariate analysis were included in the regression model, with backward elimination of the nonsignificant covariates. It was found that only LS (*β* = 0.405, *p* = 0.018) is an independent predictor of SS. The overall regression was statistically significant (*R* = 0.570, *R* ^2^ = 0.325, *F*(6, 38) = 3.05, *p* < 0.016). The results of the value inflation factors showed no evidence of multicollinearity.

**Table 3:** Correlation of SS and parameters in patients with the PVT group.

	**SS (kPa) (** **n** = 46 **)**	
	**Spearman correlation coefficient (** **r**_**s**_ **)**	**p**
LS	0.45	0.002
Spleen length	0.40	0.006
Platelet count	−0.26	0.083
Bilirubin	0.26	0.087
Age	0.15	0.328
BMI	0.01	0.971

Abbreviations: BMI, body mass index; LS, liver stiffness; SS, spleen stiffness.

### 3.8. Correlation of Anatomical Distribution of PVT With LS and SS

Patients with extensive and main trunk PVT had a higher median SS than those with branch or intrahepatic PVT (extensive: 39.6 [21.1–53.7], main: 59.1 [32.4–86.2], branch: 28.3 [19.5–35.2], intrahepatic: 19.5 [15.0–24.1] kPa, *p* < 0.005).

**Figure 4:**
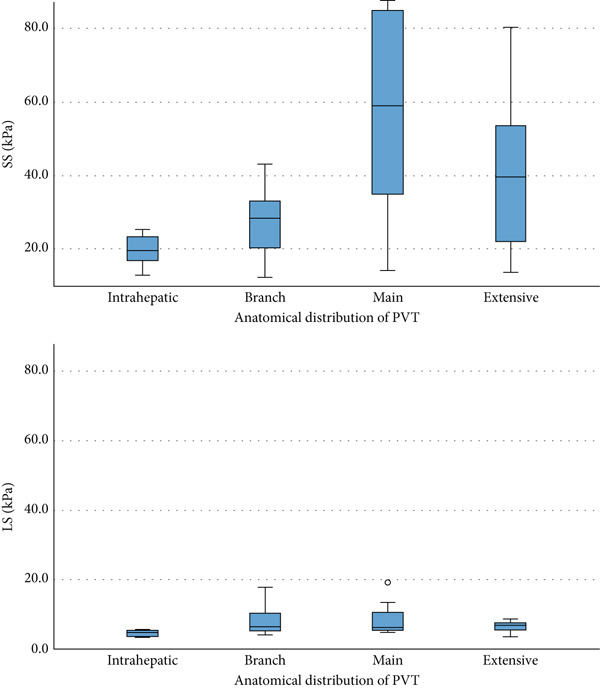
Graph showing SS and LS in different anatomical distributions. LS = liver stiffness; SS = spleen stiffness.

For LS, patients with extensive and main trunk PVT had a higher median LS than those with branch or intrahepatic PVT (extensive: 6.7 [5.4–7.6], main: 6.2 [5.2–11.9], branch: 6.4 [5.1–10.3], intrahepatic: 4.6 [3.5–5.5] kPa, *p* = 0.011) (Figure 4).

## 4. Discussion

TE is an established tool in risk stratifying cirrhosis, enabling early treatment for portal hypertension and may be used to avoid unnecessary endoscopies. To our knowledge, this pilot study is the first to investigate the correlation of SS with both PVT and PVT‐induced portal hypertension.

In this study, patients with PVT had higher SS than the control patients (27.9 vs. 16.9 kPa, *p* < 0.001). This finding of a higher SS in PVT patients is compatible with the Sharma [14] study, which compared EHPVO patients and a control group (51.7 vs. 16.0 kPa).

Higher SS may be a result of splenomegaly, splenic congestion, and remodeling that develops secondary to hyperdynamic portal circulation [21], compatible with histological reports showing hyperplasia of histiocytes and myofibroblasts, blood pooling, increased reticular fibers, and hyperplasia of arterioles in the spleen [22, 23].

This study showed lower SS values than in cirrhosis (≈ 40 kPa [24]), but prior studies [25] with EHPVO (≈ 50–60 kPa) showed higher values, likely due to the exclusion of intrahepatic and branch‐type occlusions. This reflects differences in underlying pathophysiology: In cirrhosis, sinusoidal portal hypertension is present, whereas prehepatic portal hypertension in PVT is primarily driven by the degree of occlusion, venous congestion, and splenomegaly.

The higher LS value in PVT patients compared with the control group is a surprising finding, as PVT does not induce fibrosis as in other chronic liver diseases. Huang et al. [26] reported consistently high LS (10.3–17.3 kPa) in a patient with noncirrhotic PVT, with histology confirming no evidence of fibrosis or cirrhosis. The hypothesis is that a compensatory increase in arterial perfusion occurs as a result of low portal vein flow and that portal biliopathy increases biliary pressure, which also leads to a higher LS measurement.

PVT patients with portal hypertension had higher SS (77.7 vs. 24.4 kPa, *p* < 0.001), whereas there was no statistical difference in LS when comparing between the two groups. This suggests that SS performs better than LS in identifying portal hypertension in PVT patients. However, there was a weak correlation between LS and SS (*r* = 0.446, *p* = 0.002) in PVT patients. Further studies should be done to explore the role of LS in PVT.

Patients with main and extensive involvement of PVT have higher SS (extensive: 39.6, main: 59.1, branch: 28.3 kPa, intrahepatic: 19.5, *p* < 0.001). Similarly, Gioia et al. [27] studied 79 patients with both acute and chronic noncirrhotic nonmalignant PVT. Multivariate and univariate analysis showed multisegmental PVT (along with previous variceal bleeding and large varices at basal endoscopy) as variables associated with higher risk of variceal bleeding. Although no other studies have directly demonstrated a correlation between thrombus size and SS, it is plausible that larger thrombi cause greater venous outflow obstruction, leading to increased backflow and splenic congestion. Supporting this, Yuldashev et al. [25] reported higher SS values in patients with extensive hepatic/portal vein obstruction (EHVPO) without collaterals compared with those with collaterals, suggesting that greater obstruction and venous pressure are associated with increased SS.

Higher SS in beta‐blocker users (32.9 vs. 21.8 kPa) appears counterintuitive, but adjustment for thrombosis extent (~12% reduction) suggests this reflects confounding rather than a direct effect of the drug.

These findings may carry important clinical implications. Firstly, although CT remains the gold standard for diagnosing PVT, these findings suggest that SS may offer a valuable, radiation‐free adjunct for interval monitoring of PVT progression. Its use may reduce the frequency of interval CT scans, minimizing radiation exposure, contrast‐related risks, and healthcare costs. Secondly, current guidelines recommend routine variceal screening in all patients with PVT. However, a validated tool that risk stratifies portal hypertension to spare patients from unnecessary upper endoscopy in PVT is not yet available. Although the Baveno VII consensus [28] suggests using LS (by rule‐of‐five of 10–15–20–25 kPa) in combination with platelet count (based on the ANTICIPATE model) to estimate the risk of liver‐related outcomes in cirrhosis, this is not applicable to PVT. Further research is warranted to investigate whether noninvasive modalities such as SS can reliably reflect PVT‐induced portal hypertension.

### 4.1. Limitations

This study has several limitations that warrant cautious interpretation of the findings. Firstly, as a pilot study with a small sample size, the results are exploratory and should be interpreted with caution. Secondly, not all patients underwent variceal screening in this study, and both radiological imaging and elastography have diagnostic limitations for portal hypertension. Additionally, misclassification may have occurred, as portal hypertension could have developed after the study time point. Thirdly, as TE was not performed at real‐time PVT diagnosis, there remains a potential for misclassification. Nevertheless, it is noteworthy that existing evidence indicates PVT recanalization generally occurs more than 3 months after onset [27]. Fourthly, the predominant etiology in this study was intra‐abdominal sepsis with branch or intrahepatic PVT involvement, which differs from Western epidemiological studies of PVT [29] (predominantly hematological malignancies and hereditary thrombophilia). Therefore, these findings should be interpreted with caution given the confounding influence of etiological and anatomical involvement of PVT on SS. Fifthly, the delay of SS measurement from anticoagulation initiation may introduce potential bias regarding the interpretation of elastographic parameters in relation to PVT status as partial or complete recanalization may occur within weeks following treatment. In noncirrhotic patients, early anticoagulation can yield recanalization rates of 39% at 6 months [30]. Lastly, TE has limitations compared with other modalities like shear wave elastography or magnetic resonance elastography, as it cannot select a region of interest, resulting in less reliable heterogeneous tissue evaluation. Repeated TE measurements may ensure reproducibility.

In conclusion, our pilot study suggests a potential correlation between SS and PVT and PVT‐induced portal hypertension. Further large‐scale, prospective studies are required to validate these results to determine whether SS can be integrated into the diagnostic and monitoring framework for PVT.

## Ethics Statement

This study was approved by the New Territories West Cluster Research Ethics Committee of Hospital Authority, Hong Kong.

## Conflicts of Interest

The authors declare no conflicts of interest.

## Author Contributions

**K.T.L.C.**: conceptualization, methodology, software, validation, writing—original draft. **O.L.C.**: resources. **Y.Y.L.**: investigation, resources. **S.W.L.L.**: writing—review and editing, supervision.

## Funding

No funding was received for this manuscript.

## Data Availability

The data that support the findings of this study are available from the corresponding author upon reasonable request.
